# VIGET: A web portal for study of vaccine-induced host responses based on Reactome pathways and ImmPort data

**DOI:** 10.3389/fimmu.2023.1141030

**Published:** 2023-03-21

**Authors:** Timothy Brunson, Nasim Sanati, Anthony Huffman, Anna Maria Masci, Jie Zheng, Michael F. Cooke, Patrick Conley, Yongqun He, Guanming Wu

**Affiliations:** ^1^ Department of Medical Informatics and Clinical Epidemiology, Oregon Health and Science University, Portland, OR, United States; ^2^ Department for Computational Medicine and Biology, University of Michigan, Ann Arbor, MI, United States; ^3^ Department of Biostatistics and Bioinformatics, Duke University School of Medicine, Durham, NC, United States; ^4^ Office of Data Science, National Institute of Environmental Health Sciences, Research Triangle Park, NC, United States; ^5^ Department of Genetics, University of Pennsylvania Perelman School of Medicine, Philadelphia, PA, United States; ^6^ Unit for Laboratory Animal Medicine, University of Michigan Medical School, Ann Arbor, MI, United States; ^7^ Department of Microbiology and Immunology, University of Michigan Medical School, Ann Arbor, MI, United States

**Keywords:** vaccination response, yellow fever vaccines, biological pathways, reactome, pathway analysis, differential gene expression, data visualization, web application

## Abstract

Host responses to vaccines are complex but important to investigate. To facilitate the study, we have developed a tool called Vaccine Induced Gene Expression Analysis Tool (VIGET), with the aim to provide an interactive online tool for users to efficiently and robustly analyze the host immune response gene expression data collected in the ImmPort/GEO databases. VIGET allows users to select vaccines, choose ImmPort studies, set up analysis models by choosing confounding variables and two groups of samples having different vaccination times, and then perform differential expression analysis to select genes for pathway enrichment analysis and functional interaction network construction using the Reactome’s web services. VIGET provides features for users to compare results from two analyses, facilitating comparative response analysis across different demographic groups. VIGET uses the Vaccine Ontology (VO) to classify various types of vaccines such as live or inactivated flu vaccines, yellow fever vaccines, etc. To showcase the utilities of VIGET, we conducted a longitudinal analysis of immune responses to yellow fever vaccines and found an intriguing complex activity response pattern of pathways in the immune system annotated in Reactome, demonstrating that VIGET is a valuable web portal that supports effective vaccine response studies using Reactome pathways and ImmPort data.

## 1 Introduction

As one of the most significant inventions in modern medicine, vaccination has been used to dramatically protect humans against many infectious diseases and improve human health. However, our efforts to develop vaccines to protect against many diseases have not always been successful. Future success of effective vaccine development relies on a deep understanding of the molecular mechanisms of vaccine-induced host responses including host molecular interactions and pathways, and must be based on more powerful tools to support rational vaccine design.

Biological pathways are concepts annotated for specific functions carried out by the series of biochemical reactions involving multiple molecules, each of which plays roles as inputs, outputs, catalysts, activators, or inhibitors in reactions. Pathway-based approaches are commonly used for large scale omics data analysis for researchers to learn the functional context and molecular mechanisms underlying the biological problems. One complexity with pathway-based functional analysis is pathway crosstalk because many proteins may have significant roles in multiple pathways. To mitigate this complexity, cellular level networks are frequently constructed to place significant entities in a single holistic view to avoid defining pathway boundaries and reveal crosstalks among overlapped pathways. Reactome (http://www.reactome.org) (1) is the most comprehensive open source, manually curated biological pathway knowledgebase, widely used in the community for pathway-based data analysis and visualization. Many vaccine-related pathways have been annotated in Reactome, including pathways in Immune System (https://reactome.org/PathwayBrowser/#/R-HSA-168256), infectious diseases (https://reactome.org/PathwayBrowser/#/R-HSA-5663205), and diseases of immune system (https://reactome.org/PathwayBrowser/#/R-HSA-5260271), among many other signaling pathways and biological processes. For genome-scale network-based data analysis and holistic visualization, Reactome provides a highly reliable functional interaction network, called “Reactome FI network”, covering over 60% of total human protein-coding genes, by extracting interactions from manually curated pathways and predicting interactions based on a machine learning technique (2).

ImmPort (the Immunology Database and Analysis Portal; https://www.immport.org) is the world’s largest repository of public-domain, de-identified clinical trial data related to immunology (3, 4). All data derived from clinical trials funded by the Division of Allergy, Immunology and Transplantation (DAIT) of the National Institute of Allergy and Infectious Diseases (NIAID) are required to be published on the ImmPort portal. ImmPort includes complete clinical and mechanistic study data, all of which are publicly available for downloading in a de-identified form. To support data integration, ontologies have been used for representation of various data types in ImmPort. For example, the Vaccine Ontology (VO) (5) has been used for representation of vaccines reported in ImmPort. Very recently ImmPort has integrated a suite of analysis and visualization tools for users to perform data analysis directly on the web site. However, in order to use these tools more efficiently, some familiarity with R or Python programming is required.

In this paper, we describe a web-based data analysis portal for vaccine response studies called “VIGET” for ***V*** accine ***I*** nduced ***G*** ene ***E*** xpression Analysis ***T*** ool. The portal collected all vaccination studies hosted at ImmPort, pre-processed gene expression data after downloading them from Gene Expression Omnibus (GEO, https://www.ncbi.nlm.nih.gov/geo/), and offers a suite of streamlined JavaScript-based user interfaces. Users of the portal can conduct differential gene expression analysis on the fly, perform pathway enrichment analysis and build a functional interaction network *via* a seamless integration with the Reactome’s web application, facilitating the understanding of the molecular mechanisms of vaccine response for patients with a variant of demographic background *via* comparison analysis.

## 2 Methods

### 2.1 Collection and annotation of vaccine response data

**Figure 1:**
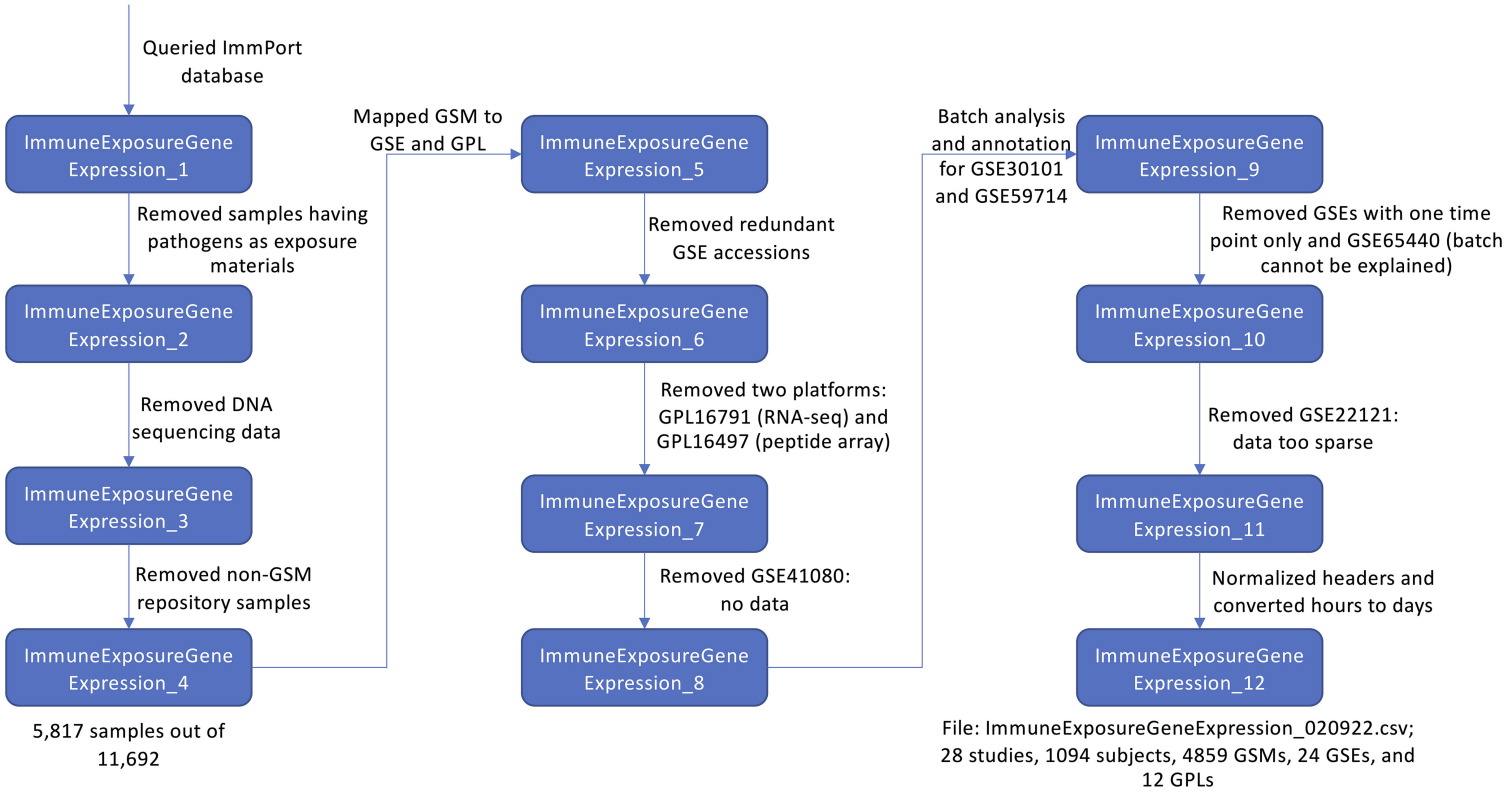
Flowchart of the workflow on collecting vaccination metadata from ImmPort and manual checking and annotation.

We downloaded the ImmPort MySQL database from the ImmPort’s download site (Version available in September, 2020) and used an in-house developed workflow (**Figure 1**) to generate a meta file for vaccine response data collected in ImmPort *via* scripting and manual checking and annotation. Briefly, we queried the downloaded ImmPort MySQL database *via* a Hibernate API (https://hibernate.org, version 5.2.11) to collect all ExpSample objects (https://www.immport.org/shared/dataModel) and their related information focusing on subjects having information of immune exposure with gene expression (output in ImmpuneExposureGeneExpression_1). To focus our data on vaccination study, we removed rows having pathogens as exposure materials, which do not have VO annotated vaccine identifier records (ImmuneExposureGeneExpression_2). Further, we removed rows for DNA sequencing data (ImmuneExposureGeneExpression_3) and kept only samples having GSM accession numbers (ImmuneExposureGeneExpression_4). After these three filtering processes, we gathered 5,817 samples out of 11,692 originally collected at the ImmPort database. To collect gene expression data directly from GEO, we mapped GSM accession numbers to GSE and GPL using the GEOmetadb.sqlite database embedded in R package, GEOmetadb (6) (version 1.48.0) (ImmuneExposureGeneExpression_5). Some GSM records may be referred to by more than one GSE record. To avoid duplication, we removed redundant GSE accessions by following the two rules after conducting a pairwise overlapping analysis (ImmuneExposureGeneExpression_6): (i) In ImmuneExposureGeneExpression_5, if both GSE accessions contained the same number of GSM accession numbers, we kept the GSE accession having the smaller number (e.g., GSE13486 and GSE13485 both contain 87 GSM accessions. We kept GSE13485 to minimize the number of samples in the downloaded gene expression matrix file); (ii) If one GSE accession covers all GSM accessions contained in another GSE accession, the second GSE accession was removed. Our current analysis platform supports gene expression data generated by microarray platforms only, therefore we removed data generated from two platforms (ImmuneExposureGeneExpression_7): GPL16791 (RNA-seq) and GPL16497 (peptide array). GSE41080 does not contain gene expression data in the matrix format, so we removed all samples in this dataset (ImmuneExposureGeneExpression_8).

Based on GSE accessions in ImmuneExposureGeneExpression_8, we wrote an R script (processor.R, https://github.com/VIOLINet/immport-ws/blob/master/src/main/resources/data_eng/processor.R) to download the pre-processed gene expression data in the matrix files from GEO, conducted a PCA analysis for batch effect inspection for individual GSE datasets, and then annotated each dataset manually based on the batch effect analysis results (**Table S1** in **Supplemental Material**) by adding a new batch column to the ImmuneExposureGeneExpression file (ImmuneExposureGeneExpression_9). Since the focus of our platform is for differential expression analysis, we removed GSE accessions having only one vaccination time point. Also, we found a batch effect in GSE65440 that could not be explained based on the metadata provided in GEO and ImmPort. Therefore, we removed GSE65440 (ImmuneExposureGeneExpression_10). Based on the PCA analysis and manual inspection, we found the data in GSE22121 is too sparse with missing values for many genes and samples and thus removed it (ImmuneExposureGeneExpression_11). To integrate the metadata file seamlessly with the Vuejs-based web frontend app, we further normalized the headers and converted hours to days (ImmuneExposureGeneExpression_12). In total, the final file (ImmuneExposureGeneExpression_020922.csv) covers 28 studies, 4,859 GSM accessions collected from 24 GSE records and generated from 12 GPL platforms for 20 vaccines based on VO identifiers.

To generate a single file for gene expression covering all samples annotated in the above ImmuneExposureGeneExpression file, we mapped probe sets to gene symbols based on the “GENE SYMBOL” or “SYMBOL” column in the GPL annotation files downloaded from GEO. For GPL annotation files have neither of these two columns, we used an UniGene file (Hs.data.gz, generated on April 25, 2013) downloaded from https://ftp.ncbi.nlm.nih.gov/repository/UniGene/Homo_sapiens/, and extracted three fields, UniGene, GENE_ID (i.e. LOCUSLINK) and Gene Symbol to map UniGene (GPL7567), GENE_ID (GPL10647), or LOCUSLINK (GPL9700 and GPL10465) to gene symbols. We dropped probe sets that could be mapped to more than one gene symbol and used the median values if multiple probe sets could be mapped to one single gene symbol. After mapping probe sets to gene symbols for individual expression matrix files, we merged all data together into one single CSV file using the DataFrame’s join function in pandas, a Python package (https://pandas.pydata.org). To normalize all gene symbols to the latest officially approved human gene symbols, we generated a mapping file *via* https://www.genenames.org/download/custom/ after selecting the following fields: HGNC ID, Approved symbol, Approved name, Status, Previous symbols, Alias symbols, Chromosome, Accession numbers, RefSeq IDs, NCBI Gene ID (downloaded in May, 2021) and mapped all outdated symbols and synonyms to the current official gene symbols. We used the median value if an official gene symbol could be mapped to more than one row. Our final gene expression matrix file covers 22,343 genes and 4,859 GSM accession numbers.

#### 2.2 Implementation of VIGET

**Figure 2:**
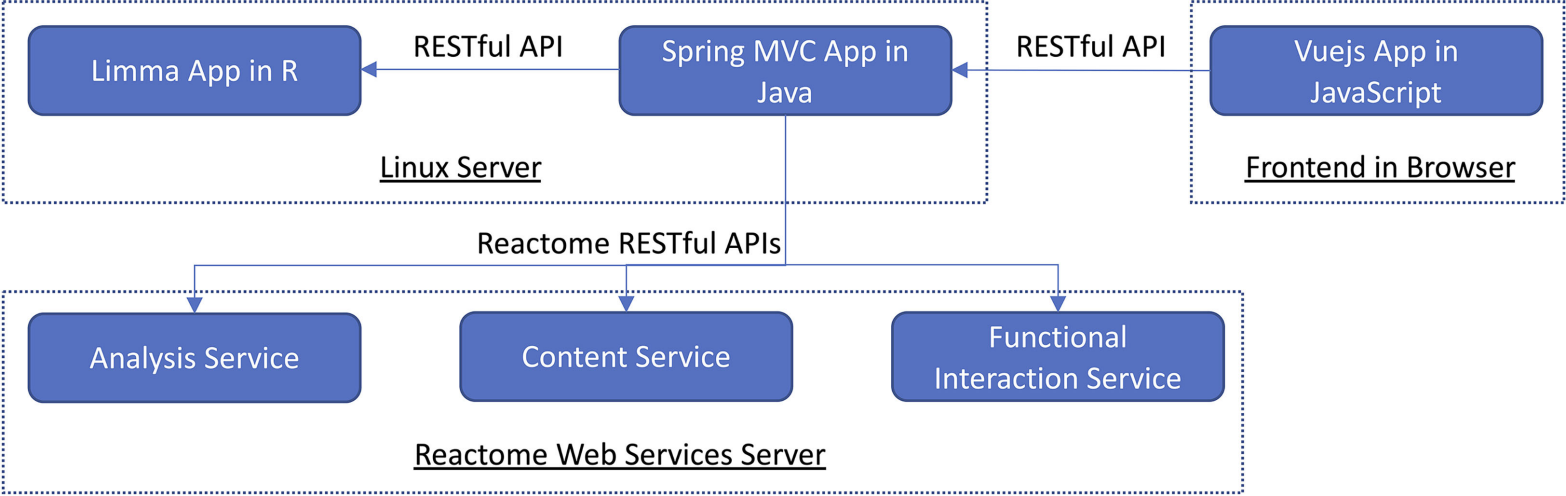
The software architecture of VIGET is composed of two tiers and three components that communicate *via* RESTful APIs.

To implement VIGET, we adopted a two-tiered software architecture composed of three components (**Figure 2**). The web frontend tier was implemented using Vuejs (version 2, https://vuejs.org) supported by several Vuejs plugins, including vuetify.js (https://vuetifyjs.com) for user interfaces, vue-cytoscape (https://rcarcasses.github.io/vue-cytoscape/), a vuejs wrapper for cytoscape.js (7) for functional interaction network view, and vue-plotly (https://github.com/David-Desmaisons/vue-plotly), a wrapper for plotly.js (https://plotly.com) for JavaScript-based volcano plot and pathway analysis result scatter plot. The server-side application was implemented as two components: a Java-based RESTful API component implemented based on the Spring web module-view-controller (MVC) framework (https://spring.io, version 4.3.10) and an R-based analysis component based on the Limma package (8) (version 3.50.1) for differential gene expression analysis. The R component was wrapped by plumber (https://www.rplumber.io, version 1.1.0) to provide a RESTful API and controlled by the Java component to start and stop. To increase the performance, the gene expression matrix was cached in the R component after the first analysis was invoked by a user. The three components communicate *via* RESTful APIs: axios (https://axios-http.com) is used between the Vuejs app and the Java component, and commons-httpclient (https://hc.apache.org/index.html, version 3.1) between the Java component and the R component. The two server-side components are hosted at a virtual server powered by RedHat 7.9 with 15.5GB RAM and 4 CPUs.

To support pathway enrichment analysis and network visualization of differentially expressed genes, we integrated Reactome’s RESTful API based web services into VIGET *via* the Java component (**Figure 2**). We integrated the analysis service (https://reactome.org/AnalysisService/) (9) for pathway enrichment analysis, the content service (https://reactome.org/ContentService/) (10) to plot the analysis results, and the functional interaction service (https://reactome.org/tools/reactome-fiviz) (11) to construct the FI network for genes. The Reactome analysis service uses the Binomial test for pathway enrichment analysis and corrects the p-values *via* the Benjamini-Hochberg false discovery rate (FDR) procedure (9).

### 2.3 Ontological representation of vaccines and variables

ImmPort provided the raw metadata for 28 studies we collected. Each of the vaccines that were used in these studies was mapped to the corresponding Vaccine Ontology (VO) term. Once done, each of the VO terms was extracted from VO to construct two hierarchies based on prior annotations: one based on vaccine target and the other by vaccine type. The vaccine target is based on NCBITaxon clades (https://www.ebi.ac.uk/ols/ontologies/ncbitaxon) for the target pathogen. The vaccine type was determined using ‘has vaccine role’ relation and the listed vaccine role to determine the hierarchy. These two hierarchies were then merged into one single hierarchical view to list vaccines at VIGET.

### 2.4 Application of VIGET for a longitudinal analysis of immune responses to yellow fever vaccines

**Figure 3:**
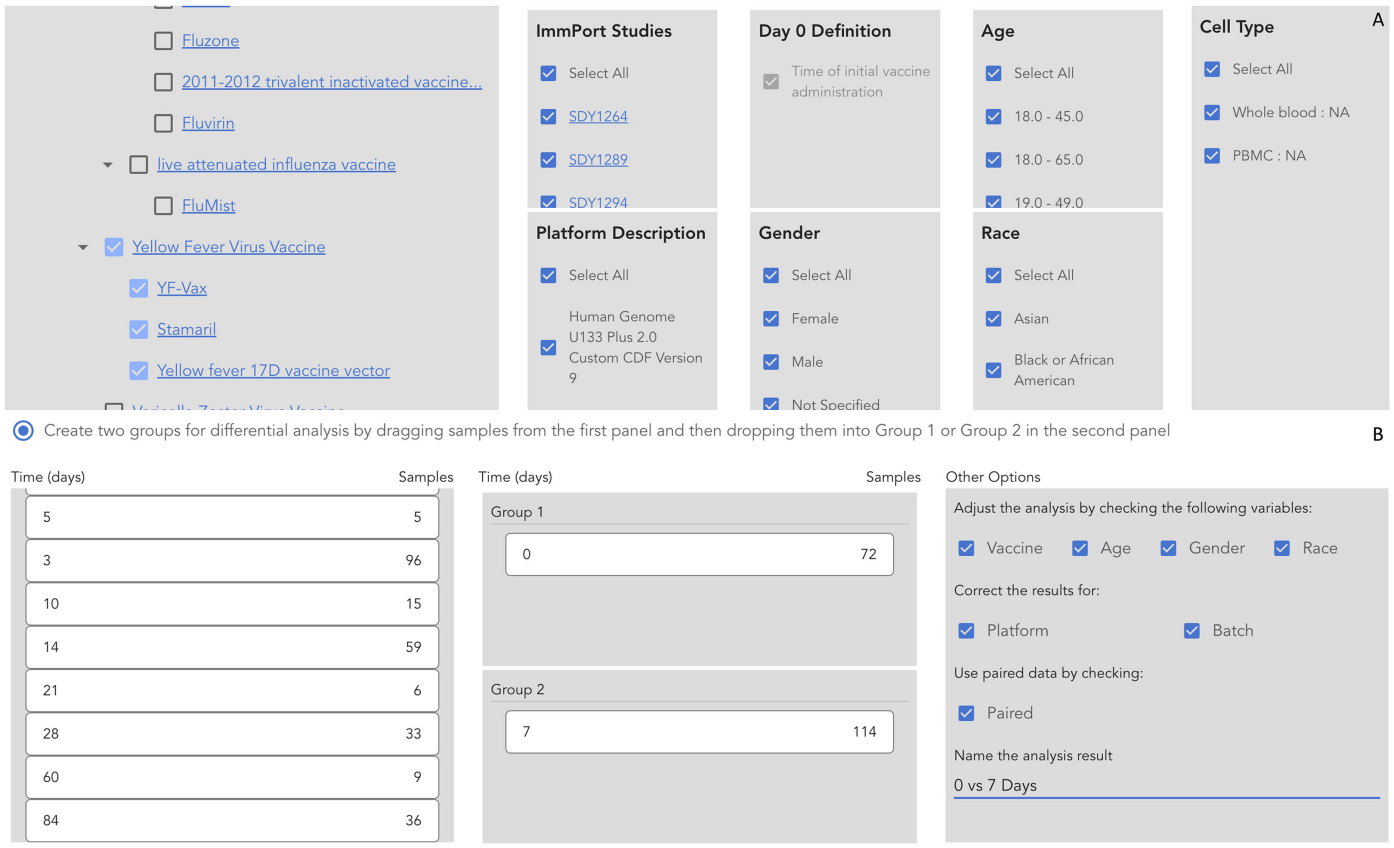
Setup parameters and the analysis model to conduct the longitudinal analysis of immune responses to yellow fever vaccines using VIGET. **(A)** Select vaccines and other parameters to select samples for the analysis. **(B)** Choose time points and select confounding variables for differential gene expression analysis.

To demonstrate the utilities of VIGET for vaccine response studies, we conducted a longitudinal analysis of the immune responses to the yellow fever vaccines. For this analysis, we checked the following parameters to select samples for analysis in VIGET (**Figure 3A**): Yellow Fever Virus Vaccine (VO_0000123) in the Vaccine panel, “Select All” in ImmPort Studies, Platform Description, Gender, Age, Race, and Cell Type. For the differential gene expression analysis, we checked vaccine, age, gender and race to adjust the limma model. Further, we also checked platform and batch for correction, and used paired samples by checking “Paired” (**Figure 3B**) to ensure only subjects having expression data in both groups contribute to the final analysis results. We analyzed samples collected at three time points post vaccination: 7 days (one week), 14 days (two weeks), and 28 days (4 weeks). To learn the immune responses at each time point, we conducted differential gene expression analysis for three pairs of time points: 7 vs 0 (days), 14 vs 0, and 28 vs 0 post vaccination. To learn the temporal responses during these time points, we also conducted the differential expression analyses for two more consecutive time pairs: 14 vs 7 and 28 vs 14 (days) post vaccination. For pathway enrichment analysis, we chose genes having log2 fold changes greater than 0.2 or less than -0.2. Some genes submitted to pathway enrichment analysis may have adjusted p-values greater than 0.05. We selected genes for pathway analysis based on log2 fold change only in order to increase the analysis sensitivity based on the hits of the immune response pathways.

## 3 Results

### 3.1 A comprehensive set of vaccine response gene expression data

To support the systematic analysis of vaccine responses for a variety of vaccines across different demographic categories, we developed a comprehensive script-based workflow and collected a large set of gene expression data from GEO based on manually annotated studies in ImmPort. The final dataset covers 21 vaccines based on vaccine names (20 based on VO term ids), 28 ImmPort studies, and 4,859 biosamples (i.e. GSM samples) that were collected in 24 GSE datasets and conducted using 12 GPL platforms. The samples in the dataset were annotated for 7 races, 4 cell types and 184 cell subtypes with ages ranging from 0 to 90 years and vaccination days from -7 days to 84 days (**Table 1**). Though we have not collected any RNA-seq based dataset yet, overall, we believe this is a comprehensive large dataset for studying vaccine response in a systematic way.

**Table 1:** Statistics of the vaccine response gene expression data collected based on ImmPort studies.

Object/Variable	Number/Value
Vaccine	21 (by names) or 20 (by VO ids)
ImmPort Study	28
Race	7
Min_Age	0
Max_Age	90
Min_Day	-7
Max_Day	84
Cell Type	4
Cell Subtype	184
Biosample (GSM)	4859
GSE	24
GPL Platforms	12

Those 21 vaccines can be classified into the following categories according to the annotation in the ImmPort studies (**Table S2** in **Supplemental Material**): influenza virus vaccine including inactivated influenza vaccine and live attenuated influenza vaccine, yellow fever virus vaccine, *Neisseria meningitidis* vaccine, and other vaccines such as HIV virus vaccine, *Mycobacterium tuberculosis* vaccine, *Streptococcus pneumoniae* vaccine and Varicella-Zoster virus vaccine. Because of the wide use of influenza vaccine, it is not unexpected that we see the dominant majority of studies are for influenza virus vaccines: 3,317 GSM samples (68% of 4,859 samples in total) annotated in 16 ImmPort studies (57% of 28 studies). Yellow fever virus vaccines, including Stamaril, YF-VAX and Yellow fever 17D vaccine, have the second largest samples: 482 GSM samples annotated in 4 studies.

**Figure 4:**
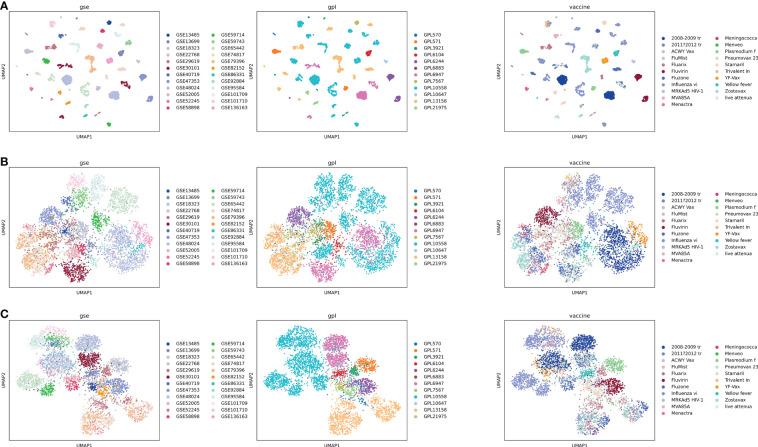
UMAP plot of all samples, colored based on vaccines, platforms, and GSEs. GSE-based batch effect is observed clearly **(A)**, and batch correction was performed using BBKNN for GPL **(B)** or GSE **(C)**. Full vaccine names for abbreviations in the legend of the right column: 2008-2009 tr: 2008-2009 trivalent influenza vaccine; 2011?2012 tr: 2011?2012 trivalent inactivated vaccine (A/California/7/09 (H1N1), A/Perth/16/2009 (H3N2), and B/Brisbane/60/2008); Influenza vi: Influenza virus vaccine; Meningococca: Meningococcal Polysaccharide Vaccine, Groups A & C, Menomune A/C; MRKAd5 HIV-1, MRKAd5 HIV-1 gag/pol/nef; Plasmodium f, Plasmodium falciparum vaccine; Pneumovax 23, Pneumovax 23 (USA); Trivalent in, Trivalent inactivated influenza; Yellow fever, Yellow fever 17D vaccine vector; live attenua, live attenuated influenza vaccine.

Since the gene expression data was collected from multiple studies and conducted by multiple platforms, batch effect was expected and observed as shown in the UMAP (Uniform Manifold Approximation and Projection) plot (12) (**Figure 4A**). We tried a batch correction algorithm used in single cell RNA-seq data analysis called BBKNN (13) to correct batch effects for GPL (**Figure 4B**) or GSE (**Figure 4C**). As shown in **Figure 4**, BBKNN removed batch effects quite efficiently though some batch effects still exist. For example, the majority of samples analyzed using platform GPL10558, which is Illumina HumanHT-12 V4.0 expression beadchip (https://www.ncbi.nlm.nih.gov/geo/query/acc.cgi?acc=GPL10558), are distributed separately from other samples even after being corrected for GPL (**Figure 4B**). For our web-based application, therefore, we annotated the gene expression data with batch information based on GSE and batch analysis (Methods), allowing users to use the original data by integrating the batch annotation directly in the limma model (8).

**Figure 5:**
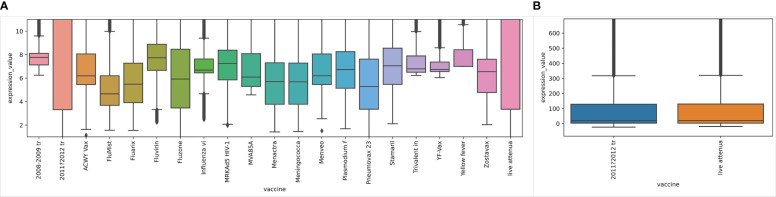
Boxplot of expression values of individual vaccines. **(A)** Zoomed-in view of the boxplot for all vaccines in the dataset. **(B)** Zoomed-in view for two vaccines having much wider distributions.

We also checked the distribution of gene expression values for individual vaccines and found that the majority of vaccines collected in our dataset have similar distributions, where expression values between 25 percentile and 75 percentile have fallen in the range between 3 and 9 (**Figure 5A**). However, two influenza vaccines, 2011-2012 trivalent inactivated vaccine (A/California/7/09 (H1N1), A/Perth/16/2009 (H3N2), and B/Brisbane/60/2008) and live attenuated influenza vaccine, have similar distributions but much wider than other vaccines: the interquartile range (IQR) is between 3 and 130 with many outliers having high expression values (**Figure 5B**).

### 3.2 A JavaScript based web application for users to conduct vaccine response analysis

**Figure 6:**
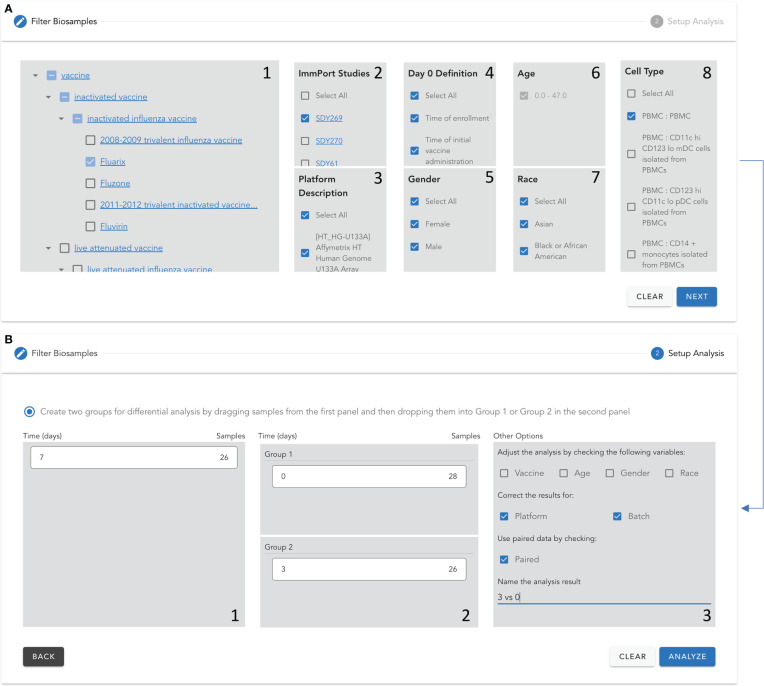
Two-step protocol to set up a differential gene expression analysis for vaccine response using VIGET. Step 1 **(A)** Select samples based on vaccine (A.1), ImmPort studies (A.2), platform used for microarray screenings (A.3), day 0 definition as annotated in ImmPort (A.4), gender (A.5), age (A.6), races (A.7) and cell types and subtypes (A.8). Step 2 **(B)** create two groups based on vaccination times listed in B.1 *via* dragging and dropping into B.2. The user can further adjust the analysis model by including confounding variables, correcting the results for platforms and choosing the paired data. Finally, the user can specify a name for the analysis results.

During the past two decades, the community has conducted tens of studies to learn vaccine responses using high throughput gene expression approaches for patients covering a variety of demographic variables with multiple platforms and data analysis models. To ease the systematic analysis of vaccine response based on these large-scale gene expression data, we developed a web-based application, called “VIGET”, by leveraging the modern JavaScript framework for users to set up analysis based on a pre-selected set of variables. This web application provides a simple two-step workflow (**Figure 6**) *via* intuitive user interfaces. Step 1 (**Figure 6A**) is designed for users to select patient samples. Users can select samples based on vaccine (A.1), ImmPort studies (A.2), platform used for microarray screenings (A.3), day 0 definition as annotated in ImmPort (A.4), gender (A.5), age (A.6), races (A.7) and cell types and subtypes (A.8). Vaccines are organized based on VO and linked to Ontobee (14) for users to browse the detailed description about listed vaccines. Similarly, ImmPort studies are listed for the selected vaccines and linked to the original study page at the ImmPort website for users to learn detailed information about each study. Step 2 (**Figure 6B**) is designed for users to choose two groups of samples for differential gene expression analysis. Users can also select confounding variables to feed into the limma model for differential expression analysis.

**Figure 7:**
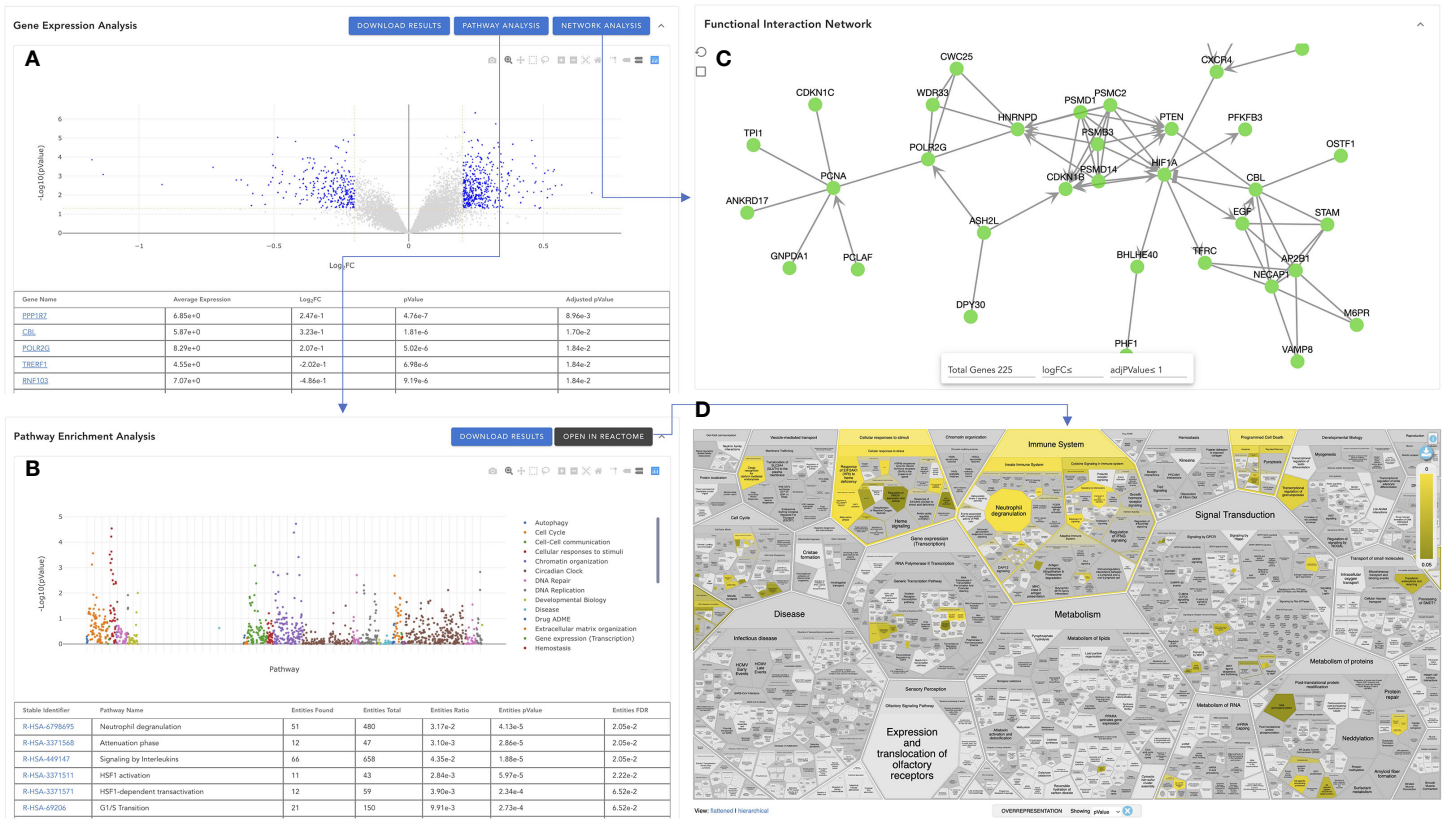
Visualization of the analysis results in VIGET. **(A)** Gene-wise differential expression analysis results widget to visualize the analysis results in a JavaScript-based volcano plot together with a table to show results for all genes. **(B)** Pathway-wise analysis results generated by Reactome’s pathway enrichment analysis for significant genes selected in A (genes plotted in blue in A). **(C)** Reactome functional interaction view of selected significant genes in **(A)**. **(D)** The pathway enrichment analysis can also be viewed directly inside the Reactome website by clicking the “OPEN IN REACTOME” button in B.

To help users visualize analysis results, VIGET provides multiple JavaScript powered interactive user interfaces (**Figure 7**). The first JavaScript widget is to show differential gene expression analysis results (**Figure 7A**), which is composed of two views: the volcano plot of logFC (log2 fold change) and -Log10(pValue) and the table view showing detailed analysis results for individual genes, where positive values of Log2FC indicate up-regulated gene expression in Group 2 vs Group 1 (**Figure 6B**). The volcano plot is provided for users to visualize all genes in a single view. Users may zoom in or out, select a specific region, or choose a specific set of genes (e.g., significantly differentially expressed genes based on chosen threshold values). In the table view, users may search for specific genes, set up thresholds based on Log2FC, pValue or Adjusted pValue. Based on the selected genes that are shown in the table and plotted in blue in the volcano plot, users may perform pathway analysis by clicking the “PATHWAY ANALYSIS” button or build a functional interaction network (15) by clicking the “NETWORK ANALYSIS” button.

Reactome is the most comprehensive, open source, open access biological pathway knowledgebase (1). We leverage the pathway analysis feature of Reactome by directly submitting the selected genes *via* its RESTful API (https://reactome.org/AnalysisService/). The pathway analysis results are displayed in the “Pathway Enrichment Analysis’’ widget (**Figure 7B**). Similar to the gene expression analysis widget, the pathway widget provides two views: the scatter plot view and the table view. The scatter plot shows Reactome pathways at the x-axis and -log10(pvalue) at the y-axis. To facilitate the comparison between different analyses, we fix the order of the Reactome pathways at the x-axis according to the pathway hierarchical organization in Reactome. Pathways are colored based on their top-level pathway assignments as annotated in Reactome. Users can choose one or more specific top-level pathways, such as “Immune System” and “Signal Transduction”, for zoomed-in view. The table view displays the detailed analysis results returned from the Reactome analysis web service, where users can search for pathways, and filter displayed pathways based on pValue or FDR (False Discovery Rate). The pathway widget also provides a link, “OPEN IN REACTOME”, for users to browse the analysis results directly inside the Reactome’s website *via* its Voronoi map (16) (**Figure 7D**), a detailed holistic view of all Reactome pathways. Users may click the pathway links in the table to view the analysis results at Reactome for individual pathways.

Proteins or genes are organized in pathways for better understanding their biological functions. However, because of the difficulty of setting the pathway boundaries and learning the crosstalk between pathways, network-based approaches are preferred to learn functional relationships among genes or proteins. VIGET uses the Reactome functional interactions (FIs) to construct a FI network for the selected significant genes. The FI network widget (**Figure 7C**) offers users to study potential functional relationships, where users can select genes for display based on logFC and adjPValue (adjusted p-value) or total number of genes in the network. The user may perform network clustering to better know the organization among genes in the network (11).

**Figure 8:**
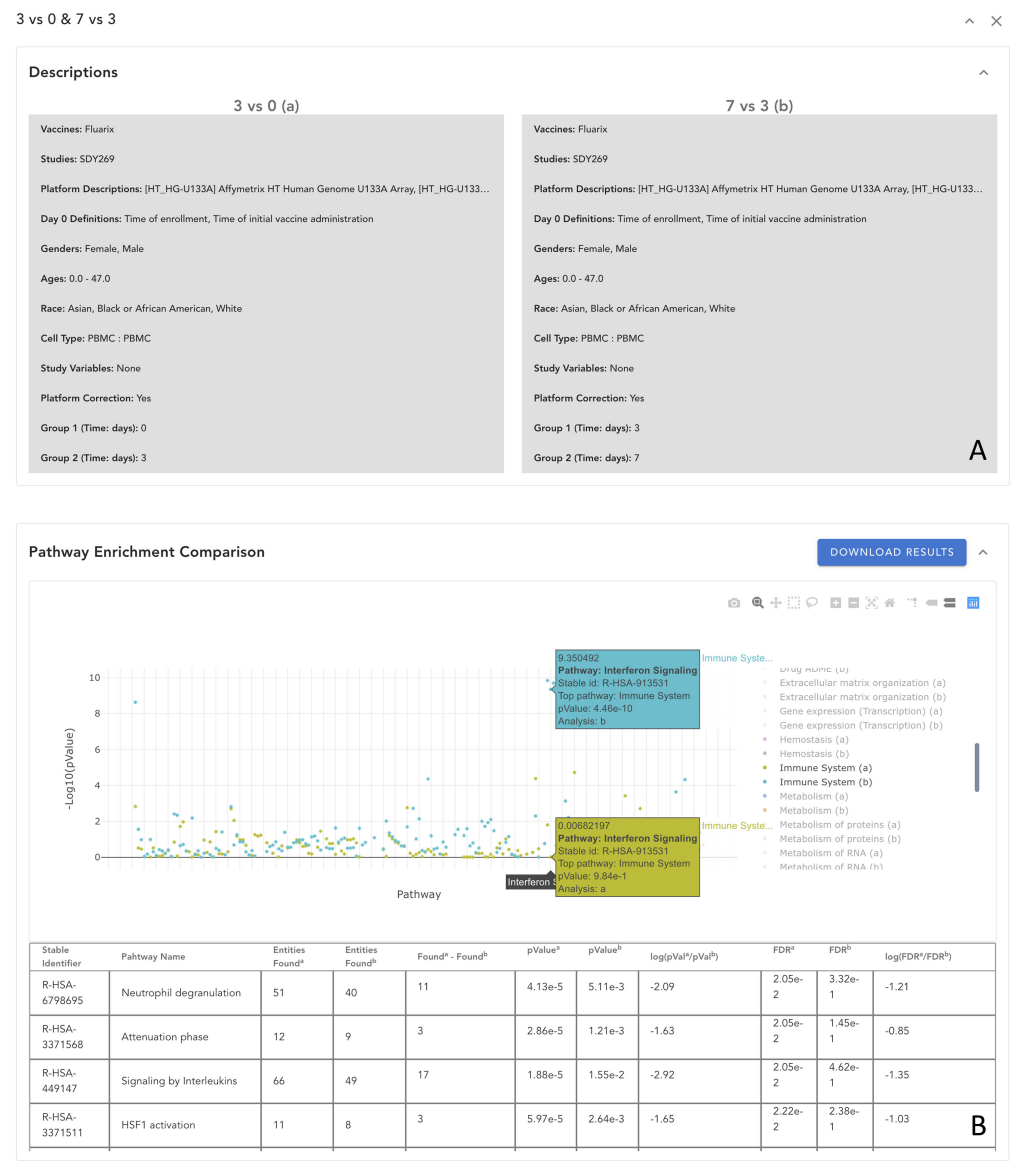
Comparison of analysis results in VIGET. A comparison analysis was set up in VIGET for two analyses in **(A)** between vaccine response on day 3 and day 7 post vaccination by Fluarix (an influenza vaccine) collected in study SDY269. The user can compare results in a collective view such as the pathway plot view shown in **(B)** where only pathways annotated in “Immune System” are shown for simplicity. As we can see in this pathway comparison, the first three days have stronger responses in the “Signaling by Interleukin” pathways as shown in the bottom table. However, after three days, the “Interferon Signaling” pathways become stronger as marked in the plot. The user can also compare results directly in the volcano plot and the network view (not shown here).

Understanding the temporal response of vaccination and the differential immune response of patients from different demographic groups are imperative to better understanding the vaccination molecular mechanisms and design better vaccines. These needs can be addressed based on comparison analyses. VIGET provides a simple user interface for users to conduct a comparison study between two analyses, such as response between 3 days vs 0 days and 7 days vs 3 days (**Figure 8A**) and then visualize comparison results in the enhanced differential expression widget, pathway widget, and network widget. For example, **Figure 8B** shows the enhanced pathway widget displaying the comparison results between 3 days vs 0 days (a) and 7 days vs 3 days (b) zoomed into pathways annotated under “Immune System” in Reactome. This comparison analysis results implied that around 7 days the interferon signaling pathway activity has a significant change while earlier days (i.e., 3 days) the signaling by interleukins pathway activity does so.

### 3.3 Application of VIGET to vaccine response study

To showcase the utilities of VIGET for vaccine response studies, we conducted a longitudinal analysis of immune responses to yellow fever vaccines. As noted above, VIGET collected multiple longitudinal data for a variety of vaccines, providing researchers a great opportunity to study temporal immune response *via* adjusting parameters interactively and then conducting differential gene expression analysis on the fly. In Reactome, pathways in immune systems are divided into three categories: adaptive immune system, innate immune system and cytokine signaling in immune system (https://reactome.org/PathwayBrowser/#/R-HSA-168256). Utilizing the data collected in VIGET, we conducted a longitudinal study of immune responses to yellow fever vaccines by investigating significant pathways in these three categories and other related pathways in Reactome. For yellow fever vaccines, VIGET imported 4 ImmPort studies, covering three types of yellow fever virus vaccines, including YF-Vax (VO_0000121), Stamaril (VO_0003139), and Yellow fever 17D vaccine vector (VO_0000122), for 482 subject samples collected from either whole blood or PBMCs (peripheral blood mononuclear cells). These subjects ranged from 18 years old to 65 years old, including Asian and African American, and males and females. These samples covered post vaccination time from day 0 to day 84, providing a comprehensive dataset to study longitudinal vaccination responses.

**Figure 9:**
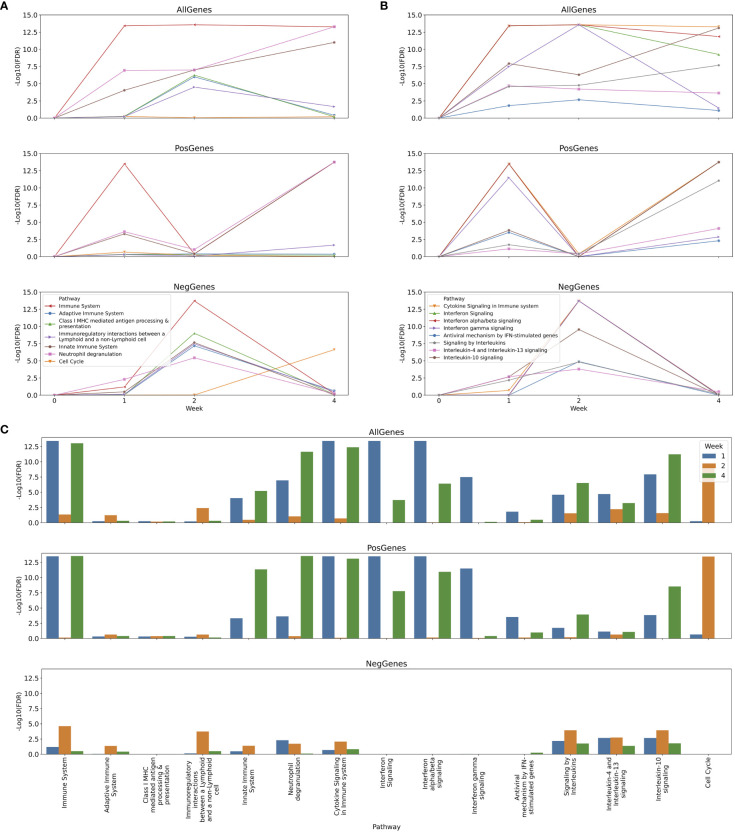
Longitudinal analysis of immune responses to yellow fever vaccines at one, two and four weeks post vaccination. **(A)** Temporal changes of major immune response pathways and cell cycle; **(B)** Temporal changes of cytokine signaling pathways during the first four weeks post vaccination; **(C)** Immune response pathways enriched for genes significantly differentially expressed at one, two and four weeks post vaccination. In **(A, B)** the differential gene expression analysis was conducted between two consecutive time points (e.g. between one week and two weeks), while in C the analysis conducted between the indicated week vs 0 week post vaccination. All analyses were conducted three times for all differentially expressed genes, up-regulated genes and down-regulated genes, respectively, to infer directions (i.e. up or down) of pathway activity changes.

In this use case study, we focused on changes of immune pathways in the first 4 weeks (**Figure 9**; **Tables S3**-**S10** in **Supplemental Material**). The longitudinal analysis results show complicated behavior of pathways across 3 categories of immune systems in Reactome during the first 4 weeks post vaccination of yellow fever vaccines and reveal an order in which pathways are regulated. After one week post vaccination, interferon signaling (https://reactome.org/PathwayBrowser/#/R-HSA-913531), including both Interferon alpha/beta and Interferon gamma signaling pathways, were activated (**Figures 9B, C**), presumably responsible for the up-regulated activity of the Immune System top pathway (**Figures 9A, C**). During the first week the activity of Innate Immune System was also upregulated modestly, most likely resulting from Neutrophil degranulation (**Figures 9A, C**), which is annotated as a sub-pathway of Innate Immune System (https://reactome.org/PathwayBrowser/#/R-HSA-6798695). After two weeks, intriguingly, activities of all major immune response pathways were reduced compared to the first week (**Figures 9A, B**, NegGenes). However, interestingly, we saw the activity of the Cell Cycle pathway (FDR = 3.62E-14) and other related pathways (e.g. Cell Cycle, Mitotic with FDR = 3.62E-14 and Unfolded Protein Response (UPR) with FDR = 3.50E-05) were up-regulated compared to week 0 (**Figure 9C**; **Table S6** in **Supplemental Material**), implying a step of cellular preparation for the next wave of immune responses. Indeed, from two weeks to four weeks, a variety of immune response pathways increased their activities (**Figures 9A, B**, PosGenes; **Table S9** in **Supplemental Material**): Neutrophil degranulation (FDR = 1.80E-14) in Innate Immune System (FDR = 1.80E-14), Interferon alpha/beta signaling (FDR=1.80E-14) in Interferon Signaling (FDR = 1.80E-14), Interleukin-10 signaling (FDR = 1.80E-14) in Signaling by Interleukins (FDR = 8.89E-12), and Cytokine Signaling in Immune system (FDR = 1.80E-12), resulting in an overall strong Immune System (FDR = 1.80E-12) response. Interestingly, the Cell Cycle pathway (FDR = 2.38E-7) and other related pathways (e.g. Cell Cycle, Mitotic with FDR = 4.19E-9 and Unfolded Protein Response (UPR) with FDR = 3.11E-08) were reduced during the same time period (**Figure 9A**, NegGenes; **Table S10** in **Supplemental Material**) to the baseline at week 0 (**Figure 9C**; **Table S5** in **Supplemental Materials**, FourWeek_AllGenes_Pathways).

## 4 Discussion

We describe our development of the web-based VIGET tool for users to systematically analyze vaccine-induced gene expression profiles, pathways and networks on the fly with the ImmPort data and Reactome pathways. We also demonstrate the utilities of VIGET by describing a longitudinal analysis of the immune responses to yellow fever vaccines using the Reactome pathways annotated for the immune system. Our analysis unraveled the dynamic pattern of immune responses to yellow fever vaccines, showing ups and downs of some immune response pathways (e.g., cytokine signaling pathways, **Figure 8**) during the first one month post vaccination.

There are other community resources dedicated to vaccination responses. VaximmutorDB (17) is a web-based database system composed of manually curated vaccine immune factors (called “vaximmutors”), mainly genes or proteins reported in peer-reviewed articles based on differential gene expression analysis or other similar experiments. VaximmutorDB provides a comprehensive list of experiment-validated genes or proteins that are potentially underlying vaccination responses. However, the results there are pre-generated by different groups using a variety of experiments and analysis models with potentially different parameters and thresholds. Similar to VaximmutorDB, the HIPC (Human Immunology project Consortium) Dashboard (http://www.hipc-dashboard.org/) collects immune signatures from literature for the community to access and query *via* a web-enabled application (18). Contrary to VIGET, users of VaximmutorDB and the HIPC Dashboard are not able to re-analyze the original data by trying different analysis models with different parameters. Very recently, HIPC released a new web-based app called “AnalyteExplorer” (https://www.immunespace.org/project/AnalyteExplorer/begin.view) as a module at ImmuneSpace, allowing users to visualize temporal expression of a query gene in response to a set of pre-collected vaccines and pathogens and conduct enrichment analysis of the blood transcription modules (BTM) (19) for each individual study. However, AnalyteExplorer cannot combine studies or perform network-based analyses. Furthermore, the BTM modules used in AnalyteExploer are network-based modules and are not updated regularly while Reactome pathways used in VIGET are based on manually curated temporal and causal relationships among biochemical reactions and are updated periodically (every three months).

While VIGET offers a rich set of features for vaccination data visualization and analysis, it still has some limitations. The greatest issue results from the gene expression data we collected. The data was gathered from multiple studies for many vaccines, generated by a variety of microarray platforms, covering various demographic variables (e.g., gender, age, and race), and collected from a great number of cell types or subtypes. Though extreme effort has been used to normalize the data and VIGET also provides interfaces for users to control these confounding variables in the limma model for robust differential gene expression analyses, care should still be taken to check the analysis results (e.g., p-values and FDRs) to ensure the results make sense statistically and biologically. Furthermore, the inconsistent time points collected from different studies limit longitudinal and comparison analyses for many vaccines. While most studies have measurements at days 3, 7, or 14 post vaccination, other time periods are inconsistent. Studies can have their final time measurement at 4 weeks (28 days), 1 month (30 days), or after 10 weeks (70 days) post vaccination. This would introduce an additional confounding variable when attempting to combine multiple days that are next to each other. Another major limitation comes from using age ranges to annotate studies in ImmPort. The effect of age on immune response provides a hurdle for analysis due to the overlapping ranges in the studies, which, however, may be mitigated with more detailed annotation to ImmPort studies that have been loaded into VIGET.

To perform pathway enrichment analysis, VIGET requires users to choose certain thresholds (e.g., log2 fold change, p-value or adjusted p-value) to select a list of genes to upload to the Reactome pathway analysis service. Usually, genes having adjusted p-value < 0.05 should be chosen. In practice, to increase the analysis performance, users may choose different threshold values (e.g., log2 fold change > 0.2 or < -0.2 as we did in our yellow fever vaccine longitudinal use case study), which requires some trial and error. To avoid this, we plan to integrate the GSEA approach in the future updates of VIGET as we demonstrated previously (20).

In summary, we have developed a web-based tool for users to conduct vaccination response studies on the fly. With the semi-automatic workflow we have developed to aggregate vaccine immune response gene expression data collected at ImmPort and GEO databases, we may update our backend dataset periodically. With the framework and the powerful web-based data analysis and visualization features in place, expansion of VIGET to include new data types such as bulk and single cell RNA-seq data, and new studies such as COVID-19 vaccine studies in ImmPort and other data repositories (e.g., GEO), represents a great opportunity to better understand patterns in vaccine immune response.

## Data availability statement

The original contributions presented in the study are included in the article/**Supplementary Material**. Further inquiries can be directed to the corresponding authors.

## Author contributions

TB, NS, AH, MC and GW developed the software. NS, PC and GW collected the data and developed the analysis tool. AH, YH and GW conducted the use case studies. AH, AM, JZ, YH performed the ontology annotation and tested the software. AH, AM, JZ, YH and GW wrote the paper. All authors discussed the results and reviewed the manuscript. YH and GW secured the funds, concept the project and supervise its implementation. All authors contributed to the article and approved the submitted version.

## Supplementary material

Supplementary Table 1 GSE gene expression annotation results after batch analysis.

Supplementary Table 2 Detailed information about individual vaccines that are arranged based on the vaccine ontology. Note: Some of the studies may cover multiple vaccines and therefore may be counted for more than one vaccine (or row) in this table.

Supplementary Table 3 Differential gene expression analysis results between one week, two weeks and four weeks post vaccination vs. 0 week, respectively.

Supplementary Table 4 Differential gene expression analysis results for one week vs 0 week, two weeks vs one week and four weeks vs two weeks.

Supplementary Table 5 Reactome pathway enrichment analysis results for genes having Log2FC > 0.2 or Log2FC < -0.2 reported in **Table S3**.

Supplementary Table 6 Reactome pathway enrichment analysis results for genes having Log2FC > 0.2 reported in **Table S3**.

Supplementary Table 7 Reactome pathway enrichment analysis results for genes having Log2FC < -0.2 reported in **Table S3**.

Supplementary Table 8 Reactome pathway enrichment analysis results for genes having Log2FC > 0.2 or Log2FC < -0.2 reported in **Table S4**.

Supplementary Table 9 Reactome pathway enrichment analysis results for genes having Log2FC > 0.2 reported in **Table S4**.

Supplementary Table 10 Reactome pathway enrichment analysis results for genes having Log2FC < -0.2 reported in **Table S4**.

## References

[1] Gillespie M Jassal B Stephan R Milacic M Rothfels K Senff-Ribeiro A. The reactome pathway knowledgebase 2022. Nucleic Acids Res (2022) 50(D1):D687–D92. doi: 10.1093/nar/gkab1028 PMC8689983 34788843

[2] Wu G Haw R. Functional interaction network construction and analysis for disease discovery. Methods Mol Biol (2017) 1558:235–53. doi: 10.1007/978-1-4939-6783-4_11 28150241

[3] Bhattacharya S Andorf S Gomes L Dunn P Schaefer H Pontius J. Immport: Disseminating data to the public for the future of immunology. Immunol Res (2014) 58(2-3):234–9. doi: 10.1007/s12026-014-8516-1 24791905

[4] Shankar RD Bhattacharya S Jujjavarapu C Andorf S Wiser JA Butte AJ. Rimmport: An R/Bioconductor package that enables ready-for-Analysis immunology research data. Bioinformatics (2017) 33(7):1101–3. doi: 10.1093/bioinformatics/btw719 PMC6041747 28057685

[5] Lin Y He Y. Ontology representation and analysis of vaccine formulation and administration and their effects on vaccine immune responses. J BioMed Semantics (2012) 3(1):17. doi: 10.1186/2041-1480-3-17 23256535 PMC3639077

[6] Zhu Y Davis S Stephens R Meltzer PS Chen Y. Geometadb: Powerful alternative search engine for the gene expression omnibus. Bioinformatics (2008) 24(23):2798–800. doi: 10.1093/bioinformatics/btn520 PMC2639278 18842599

[7] Franz M Lopes CT Huck G Dong Y Sumer O Bader GD. Cytoscape.Js: A graph theory library for visualisation and analysis. Bioinformatics (2016) 32(2):309–11. doi: 10.1093/bioinformatics/btv557 PMC4708103 26415722

[8] Ritchie ME Phipson B Wu D Hu Y Law CW Shi W. Limma powers differential expression analyses for rna-sequencing and microarray studies. Nucleic Acids Res (2015) 43(7):e47. doi: 10.1093/nar/gkv007 25605792 PMC4402510

[9] Fabregat A Sidiropoulos K Viteri G Forner O Marin-Garcia P Arnau V. Reactome pathway analysis: A high-performance in-memory approach. BMC Bioinf (2017) 18(1):142. doi: 10.1186/s12859-017-1559-2 PMC5333408 28249561

[10] Fabregat A Korninger F Viteri G Sidiropoulos K Marin-Garcia P Ping P. Reactome graph database: Efficient access to complex pathway data. PloS Comput Biol (2018) 14(1):e1005968. doi: 10.1371/journal.pcbi.1005968 29377902 PMC5805351

[11] Wu G Dawson E Duong A Haw R Stein L. Reactomefiviz: A cytoscape app for pathway and network-based data analysis. F1000Res (2014) 3:146. doi: 10.12688/f1000research.4431.2 25309732 PMC4184317

[12] McInnes L Healy J. Umap: Uniform manifold approximation and projection for dimension reduction. arXiv:180203426 (2018). doi: 10.48550/arXiv.1802.03426

[13] Polanski K Young MD Miao Z Meyer KB Teichmann SA Park JE. Bbknn: Fast batch alignment of single cell transcriptomes. Bioinformatics (2020) 36(3):964–5. doi: 10.1093/bioinformatics/btz625 PMC9883685 31400197

[14] Ong E Xiang Z Zhao B Liu Y Lin Y Zheng J. Ontobee: A linked ontology data server to support ontology term dereferencing, linkage, query and integration. Nucleic Acids Res (2017) 45(D1):D347–D52. doi: 10.1093/nar/gkw918 PMC5210626 27733503

[15] Wu G Feng X Stein L. A human functional protein interaction network and its application to cancer data analysis. Genome Biol (2010) 11(5):R53. doi: 10.1186/gb-2010-11-5-r53 20482850 PMC2898064

[16] Aurenhammer F. Voronoi diagrams–a survey of a fundamental geometric data structure. ACM Comput Surv (1991) 23. doi: 10.1145/116873.116880

[17] Berke K Sun P Ong E Sanati N Huffman A Brunson T. Vaximmutordb: A web-based vaccine immune factor database and its application for understanding vaccine-induced immune mechanisms. Front Immunol (2021) 12:639491. doi: 10.3389/fimmu.2021.639491 33777032 PMC7994782

[18] Smith KC Chawla DG Dhillon BK Ji Z Vita R van der Leest EC. A curated collection of human vaccination response signatures. Sci Data (2022) 9(1):678. doi: 10.1038/s41597-022-01558-1 36347894 PMC9643367

[19] Li S Rouphael N Duraisingham S Romero-Steiner S Presnell S Davis C. Molecular signatures of antibody responses derived from a systems biology study of five human vaccines. Nat Immunol (2014) 15(2):195–204. doi: 10.1038/ni.2789 24336226 PMC3946932

[20] Haw R Loney F Ong E He Y Wu G. Perform pathway enrichment analysis using reactomefiviz. Methods Mol Biol (2020) 2074:165–79. doi: 10.1007/978-1-4939-9873-9_13 31583638

