# Supplementary material for: VIGET: A web portal for study of vaccine-induced host responses based on Reactome pathways and ImmPort data
Source: Front Immunol. 2023 Mar 21;14:1141030. doi: 10.3389/fimmu.2023.1141030 (PMC10172660; doi:10.3389/fimmu.2023.1141030)
Supplement: Supplementary file 11 [file DataSheet_1.docx]

# Project Links

- Link to the web site: <https://viget.violinet.org/>
- Links to the GitHub Repos
  - The source code for the web frontend app: <https://github.com/VIOLINet/reactome-immport-web>
  - The source code for the server-side application: <https://github.com/VIOLINet/immport-ws>
- Link to data: <https://doi.org/10.5281/zenodo.7407195>
  - ImmuneExposureGeneExpression_020922.csv: The meta file describing aggregated ImmPort studies and related vaccines, GEO information, and some manual annotation
  - Immport_vaccine_expression_matrix_mapped_merged_approved_genes_091421.csv: The aggregate gene expression data
